# Interleukin (IL)-12 and IL-18 Synergize to Promote MAIT Cell IL-17A and IL-17F Production Independently of IL-23 Signaling

**DOI:** 10.3389/fimmu.2020.585134

**Published:** 2020-11-20

**Authors:** Suzanne Cole, Janine Murray, Catherine Simpson, Remi Okoye, Kerry Tyson, Meryn Griffiths, Dominique Baeten, Stevan Shaw, Asher Maroof

**Affiliations:** UCB Pharma, Slough, United Kingdom

**Keywords:** IL-17A, IL-17F, Th17, IL-23, mucosal associated invariant T cell, innate lymphoid cell, T cell, immune-mediated

## Abstract

IL-23 is considered a critical regulator of IL-17 in Th17 cells; however, its requirement for inducing IL-17 production in other human immune subsets remains incompletely understood. Mucosal associated invariant T (MAIT) cells uniformly express retinoic acid receptor-related orphan receptor gamma t (RORγt) but only a minor population have been shown to produce IL-17A. Here we show that IL-17F is the dominant IL-17 isoform produced by MAIT cells, not IL-17A. For optimal MAIT cell derived IL-17A and IL-17F production, T cell receptor (TCR) triggering, IL-18 and monocyte derived IL-12 signaling is required. Unlike Th17 cells, this process is independent of IL-23 signaling. Using an *in vitro* skin cell activation assay, we demonstrate that dual neutralization of both IL-17A and IL-17F resulted in greater suppression of inflammatory proteins than inhibition of IL-17A alone. Finally, we extend our findings by showing that other innate-like lymphocytes such as group 3 innate lymphoid cells (ILC3) and gamma delta (γδ) T cells are also capable of IL-23 independent IL-17A and IL-17F production. These data indicate both IL-17F and IL-17A production from MAIT cells may contribute to tissue inflammation independently of IL-23, in part explaining the therapeutic disconnect between targeting IL-17 or IL-23 in certain inflammatory diseases.

## Introduction

Both IL-17 and IL-23 have established roles in the pathogenesis of multiple autoimmune diseases. While targeting the IL-17/IL-23 axis has shown impressive clinical responses in plaque psoriasis, a disconnect between the two pathways has become evident (1). IL-23 is considered a critical regulator of IL-17 but its absolute requirement for inducing IL-17 production from human immune cells is not fully understood. A recent phase 2b clinical trial in axial spondyloarthritis found that risankizumab, a potent IL-23 neutralizing antibody, showed no clinical improvements over placebo (2). In contrast, two anti-IL-17A antibodies, secukinumab and ixekizumab showed significant clinical efficacy (3, 4). This non-linear relationship between IL-17 and IL-23 extends to inflammatory bowel disease, where IL-23 blockade improves gut inflammation (5) but neutralizing IL-17A does not (6, 7). The importance of IL-23 in the generation of polyfunctional Th17 cells has been well established in humans (8, 9) and a murine model of disease (10) while evidence for IL-17A production independent of IL-23 signaling has only been demonstrated in mice (7, 11, 12).

Mucosal associated invariant T (MAIT) cells (13, 14) have been associated with several immune-mediated inflammatory diseases and are frequently linked to increased IL-17A production at the site of inflammation. MAIT cells form the largest antigen-specific alpha beta (αβ) T cell population in the human immune system (15) and recognize vitamin B metabolites presented by the MHC-related protein 1 (MR1) (16). High constitutive receptor expression of IL-18 (IL-18Rα) and promyelocytic leukemia zinc finger (PLZF) (17, 18) enables activation of MAIT cells by innate cytokines such as IL-18 and IL-12. While cytokine or T cell receptor (TCR) stimulation alone are able to activate MAIT cells to produce IFNγ, TNF, perforin and granzymes (19), studies suggest that TCR stimulation is insufficient to produce substantial amounts of IL-17A *ex vivo* (20).

The IL-17 family comprises six members: IL-17A, IL-17B, IL-17C, IL-17D, IL-17E (also known as IL-25), and IL-17F (21). Research has focused extensively on IL-17A while IL-17F has largely been ignored, most likely due to its lower relative potency. IL-17F shares 50% sequence homology with IL-17A (22) and also signals *via* the same receptor complex to induce comparable gene expression profiles (23). *In vitro* dual neutralization of IL-17A and IL-17F with the monoclonal antibody bimekizumab offered significantly greater inhibition of inflammatory genes in normal human dermal fibroblasts (NHDFs) and synoviocytes than inhibition of either cytokine alone (24). Importantly, in multiple phase 2b clinical studies, including psoriasis, psoriatic arthritis and ankylosing spondylitis, patients treated with bimekizumab showed rapid disease improvement (25) across all key clinical domains (26, 27). Clearly the contribution of IL-17F requires further evaluation.

In this study, we set out to examine the requirement for IL-23 in the regulation of both IL-17A and IL-17F production by MAIT cells.

## Methods

### Study Design

This study was performed with human blood samples from healthy individuals under experimental laboratory conditions. Blood samples were obtained from anonymous healthy donors based at UCB Celltech, Slough, UK. Blood samples from these donors were taken with informed consent under UCB Celltech UK HTA license number 12504. All donors gave written informed consent in accordance with the Declaration of Helsinki. As described in subsequent methods, mechanistic studies were performed using *in vitro* assays without randomization or blinding. The replicates indicated in figure legends represent the number of individual donors.

### Cell Isolation and Culture

Blood samples were obtained from healthy donors with informed consent. PBMCs were isolated using Leucosep^TM^ tubes. CD8+ T cells were isolated using a CD8+ T cell isolation kit (Miltenyi Biotec). CD14+ monocytes were isolated using CD14 MicroBeads (Miltenyi Biotec). Enriched ILCs were obtained using the EasySep^TM^ Human Pan-ILC Enrichment Kit (Stemcell Technologies). Purified ILC3s were obtained by initially enriching ILCs using magnetic depletion of lineage markers by staining with biotinylated antibodies to CD3, CD4, CD19, CD94, CD1a, CD11c, CD123, BDAC2, CD14, CD34, and FcεRI followed by Anti-Biotin MicroBeads UltraPure (Miltenyi Biotec). ILC3s were further purified by gating on CD45+ Lineage- CD127+ CD161+ c-kit+ cells and sorting the enriched population using a BD Bioscience FACSAria™ III Cell Sorter (San Jose). ILCs were cultured in a 96-well plate for 7 days in DMEM with 10% FCS, glutamine and Penicillin/Streptomycin with combinations of IL-2, IL-1β, and IL-23. Between 4 × 10^3^ and 9.5 × 10^3^ ILCs were cultured per well. For TCR stimulation, 96-well plates were coated with anti-CD3 (OKT3; 1 μg/mL), and soluble anti-CD28 (CD28.2; 1 μg/mL) added to cells in X-VIVO^TM^ 15 medium (Lonza). Recombinant human cytokines IL-12, IL-18, IL-1β, IL-6, IL-23, and TGFβ, or LPS were used at 10 ng/mL. For cultures without anti-CD3/CD28 stimulation, 10 ng/mL IL-2 was added as a survival factor. 1 × 10^5^ PBMCs or isolated CD8 T cells were cultured per well. Unless otherwise stated, PBMC and CD8 T cell cultures were carried out over 3 days. Neutralizing antibodies against IL-12p40 (ustekinumab), IL-12p70 (R&D #AF-219-NA), TNF (adalimumab), IFNγ (eBioscience), and MR1 (clone 26.5) were used at 10 μg/mL. The anti-IL23p19 was generated in house and used at 10 μg/mL (validation in **Supplementary Figures 1A, B**).

### Flow Cytometry (FC)

Fluorochrome-conjugated antibodies were purchased from BD Bioscience, BioLegend or eBioscience. Antibodies against CD3-BV650, CD4-BUV805, CD8-BUV395, CD161-Alexa Fluor 647, Vα7.2-BV785, IL-17A-PE, IL-17F-PerCP-eFluor 710, IFNγ-APC-eFluor 780, TNF-PEcy7, and RORγt-PE were used for phenotypic, cytokine production, and transcription factor analysis. For cell sorting, the following antibodies were used: CD3-BV421, CD8-FITC, Vα7.2-BV785, CD161-APC, CD14-APC-eFluor 780, CD56-PEcy7, CD19-Super Bright 600, CD8-PEcy7, Vα7.2-PE, and CD25-APC/Cy7. Dead cells were excluded using Zombie Aqua™ (BioLegend). Transcription factors were assessed using the Transcription Factor Staining Buffer Set (eBioscience). Surface staining and acquisition was carried out in PBS with BSA 1% and EDTA 2 mM. Intracellular staining was carried out in permeabilization buffer (eBioscience) following fixation in formaldehyde 1.6% (Thermo Scientific Piece). Intracellular cytokines were analyzed following treatment of cells with 10 ng/mL PMA, 1 μg/mL ionomycin and 10 μg/mL Brefeldin A, or 10 μg/mL Brefeldin A only. Cells were acquired on a BD Bioscience LSRFortessa™ X-20 cytometer (San Jose) and the data analyzed using FlowJo (BD Life Science–Informatics, Ashland Oregon).

### Mass Cytometry

Antibodies (**Supplementary Table 1**) were purchased pre-conjugated (Fluidigm) or conjugated in-house with metal isotopes using the Maxpar Antibody Labeling Kit (Fluidigm). Cell-ID Intercalator-103Rh (Fluidigm) was added to cells for the final 15 min of culture to exclude dead cells. Following this, cells were washed and fixed in 1.6% formaldehyde and barcoded prior to staining using the Cell-ID 20-Plex Pd Barcoding Kit (Fluidigm). Barcoded samples were combined and stained in one tube with metal-conjugated antibodies (2 µL of 0.1 mg/mL antibody stock per sample) for 2 h at room temperature. The stained sample was washed twice and resuspended in 4°C Perm Buffer III (BD) for 20 min at 4°C. Following this, cells were washed twice and resuspended in 4% formaldehyde. Cell-ID Intercalator-Ir (Fluidigm) was added at 0.05 μM for 18 h prior to acquisition. After two washes in PBS, cells were resuspended in Cell Acquisition Solution, EQ Beads added and samples filtered through a 35 μM nylon mesh for acquisition through a wide bore injector on a CyTOF^®^ 2 with Helios upgrade. Positive staining controls for cytokine production are shown in **Supplementary Figure 2**. Cells were normalized for signal intensities of EQ beads using Helios software, and analyzed using Cytobank. viSNE analysis was applied to pre-gated Vα7.2+ CD161+ MAIT cells.

### Cytokine Profiling

ELISAs were carried out on supernatants from cultured cells (frozen at -20°C) using: IL-17A Human ELISA Kit (eBioscience), Human IL-17F DuoSet (R&D), Human CXCL8 DuoSet (R&D), Human CCL2 DuoSet (R&D), IL-6 Assay Kit (Cisbio) and Human Th Cytokine Panel (13-plex) (BioLegend), as per the manufacturers’ instructions.

### Gene Expression

RNA was extracted using the RNeasy Micro Kit (Qiagen). First strand cDNA was synthesized using the SuperScript™ VILO™ cDNA Synthesis Kit (Invitrogen), and qPCR reactions set up using 10 ng of cDNA with TaqMan Fast Advanced PCR MasterMix (Applied Biosystems) and TaqMan Assays (*ACTB*: Hs01060665_g1, B2M: Hs00187842_m1, *IL-12A*: Hs01073447_m1, *IL-12B*: Hs01011518_m1, *IL-17A*: Hs00174383_m1, *IL-17F*: Hs01028648_m1). Assays were run on a QuantStudio™ 7 Flex Real-Time PCR System (Applied Biosystems).

### MAIT Cell Activation With *E. coli*

The *E. coli* strain DH5α (N.E.B.) was grown overnight to stationary phase in Luria-Bertani broth. Serial dilutions were carried out and colonies were counted on agar plates to obtain the CFU/mL. Stocks were aliquoted in single-use vials and frozen at -80°C in 50% glycerol/50% FCS until use. For activation assays, *E. coli* was defrosted, washed once in PBS, fixed in 1% formaldehyde for 5 min, and extensively washed in PBS to remove formaldehyde. Fixed *E. coli* was immediately added to PBMCs at a ratio of 25 bacteria per cell using 1 × 10^5^ cells and 2.5 × 10^6^ CFU of *E. coli* per well. Cytokine production from PBMCs was assessed following 6 h of Brefeldin A treatment.

### RNAScope

Formalin-fixed paraffin-embedded psoriatic skin tissues were sourced from National BioService (Saint-Petersburg, Russia) from two patients with psoriasis (one guttate and one generalized pustular psoriasis) and control skin was excised during ablation of a subcutaneous tumor from an individual with no concomitant disease. Tissues were sectioned at a thickness of 5μm onto Superfrost Plus Gold glass slides (Thermo Fisher Scientific) and allowed to dry overnight at 37°C. Tissue quality was assessed by performing RNAscope analysis for mRNA of the housekeeping genes peptidylprolyl isomerase B (PPIB) and RNA Polymerase II Subunit A (POLR2A).

The slides were placed on the staining rack of the Leica Bond RX and treated with the Leica Bond RX routine factory based “Bake and Dewax” protocol before being rehydrated using ethanol. Heat-induced RNA retrieval was conducted by incubation in retrieval buffer ER2 (pH 9) (Leica Microsystems [UK] Ltd) for 15 min at 95°C, followed by protease treatment for 15 min and peroxidase blocking (Advanced Cell Diagnostics) with two rinses in distilled water between pre-treatments.

The following RNAscope probes were used in this study: Duplex Negative Control Probe (bacterial gene, dihydrodipicolinate reductase, dapB (LS, cat. #320758), Duplex Positive Control Probe (Hs-PPIB, Polr2a (LS, cat. #320748), Hs‐IL17A (LS, cat. #310938), Hs-IL17F (LS, cat. #310948), Hs-KLRB1 (LS, cat. #509038), and Hs-TRAV1-2 (LS, cat. #529408).

Briefly, 20 ZZ probe pairs targeting the relevant genomic nucleoprotein genes were designed and synthesized by Advanced Cell Diagnostics. Sections were exposed to ISH target probes and incubated at 42°C for 2 h. After rinsing, the ISH signal was amplified using company-provided Pre-amplifier and Amplifier conjugated to either alkaline phosphatase and incubated with a fast-red substrate-chromogen solution or horseradish peroxidase and incubated with 3,3’-diaminobenzidine brown substrate-chromogen solution both for 10 min at room temperature. Sections were then counterstained with hematoxylin, air-dried, before mounting in VectaMount permanent mounting medium (Vector Labs).

The stained slides were scanned with a 40× super apochromat objective using an Olympus VS120-L100-W-12 slide scanner.

### Statistics

All statistical analysis was performed using GraphPad Prism 8.1.1 (GraphPad, La Jolla, CA). All statistical tests have been indicated in figure legends. A p value <0.05 was considered significant.

## Results

### MAIT Cells Are the Predominant IL-17A and IL-17F Producing Cell Subset Within CD8+ T Cells

**Figure 1:**
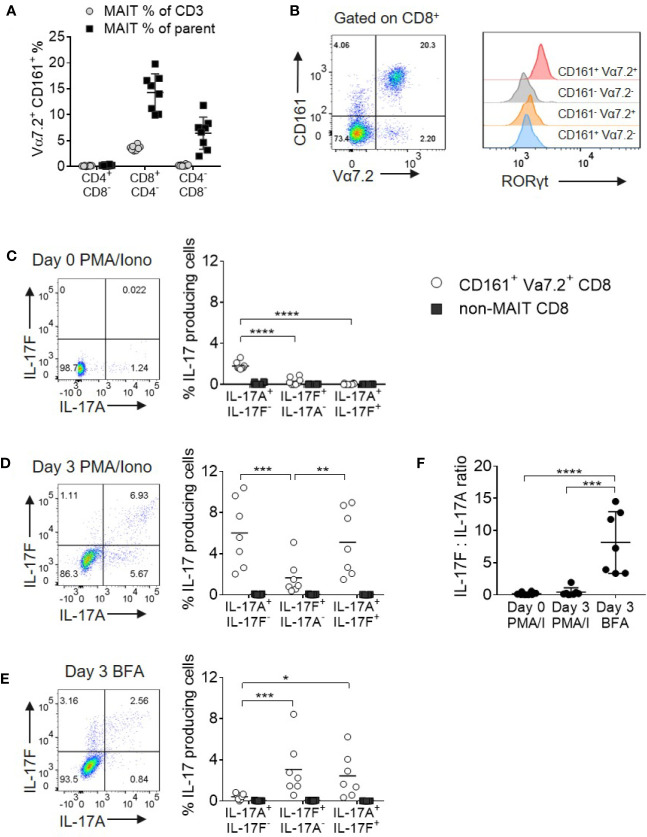
MAIT cells are the main source of IL-17A and IL-17F within CD8^+^ T cells. **(A)** Frequency of CD161^+^ Vα7.2^+^ MAIT cells from peripheral blood of healthy donors directly *ex vivo*. Frequency of MAIT cells shown either as a percentage of CD3 cells, or the parent CD4^+^CD8^-^, CD8^+^CD4^-^ or CD4^-^CD8^-^ population. *n* = 8 **(B)** RORγt expression on CD3^+^ T cell subsets from **(A)**, based on expression of Vα7.2 and CD161. **(C–E)** Representative bivariate FC plots gated on CD8^+^ MAIT cells showing the frequency of IL-17A^+^ IL-17F^-^, IL-17F^+^ IL-17A^-^, and IL-17A^+^ IL-17F^+^ producing cells. Graphs display combined results of the frequency of CD8^+^ MAIT or non-MAIT cells producing IL-17A^+^ IL-17F^-^, IL-17F^+^ IL-17A^-^, and IL-17A^+^ IL-17F^+^ following 4 h treatment with PMA/ionomycin/Brefeldin A on Day 0 **(C)** or following 3 days of activation with anti-CD3/CD28 and subsequent 4 h treatment with PMA/ionomycin/Brefeldin A **(D)** or Brefeldin A only **(E)**. The ‘non-MAIT’ cells include CD161- Vα7.2-, CD161+Vα7.2-, and CD161-Vα7.2+ cells. n = 7, statistics measured using repeated measures two-way ANOVA comparing the type of IL-17 production by the gated cell population. **(F)** Ratios of IL-17F to IL-17A in the different stimulation conditions from **(C, D)** in the same 7 donors. Statistics measured using one-way ANOVA with multiple comparisons. *p<0.05, **p<0.01, ***p<0.001, ****p<0.0001.

Given the paucity of data on IL-17F, we initially evaluated the expression of both IL-17A and IL-17F in MAIT and non-MAIT populations. MAIT cells were defined as CD161+ Vα7.2+ cells and in healthy peripheral blood, the majority were CD8+ with only minor subsets of CD4+ and CD4-CD8- cells present (**Figure 1A**). As CD8+ and CD8- MAIT cells have distinct functions (19, 28), this larger CD8+ MAIT cell subset was selected for further analysis. Resting CD8+CD161+Vα7.2+ MAIT cells were uniformly positive for RORγt when compared with their non-MAIT counterparts (**Figure 1B**); however, only a small fraction produced IL-17A after 4 h of PMA and ionomycin stimulation and no IL-17F was detected (**Figure 1C**). *In vitro* polyclonal T cell activation with anti-CD3/CD28 and subsequent PMA and ionomycin stimulation proportionally increased both IL-17A and IL-17F production (**Figure 1D**). Anti-CD3/CD28 stimulation without further PMA and ionomycin activation led to a more biased profile toward IL-17F production (**Figures 1E, F**). The only CD8+ cells producing IL-17A and IL-17F under all stimulation conditions tested were those expressing the CD161+Vα7.2+ MAIT phenotype (**Figures 1C–E** and **Supplementary Figure 3**).

As PMA and ionomycin created an artificial bias toward greater IL-17A production, we opted to examine native cytokine production using Brefeldin A only in all subsequent experiments.

### MAIT Cell IL-17A and IL-17F Production Is Enhanced Upon IL-12 and IL-18 Signaling

**Figure 2:**
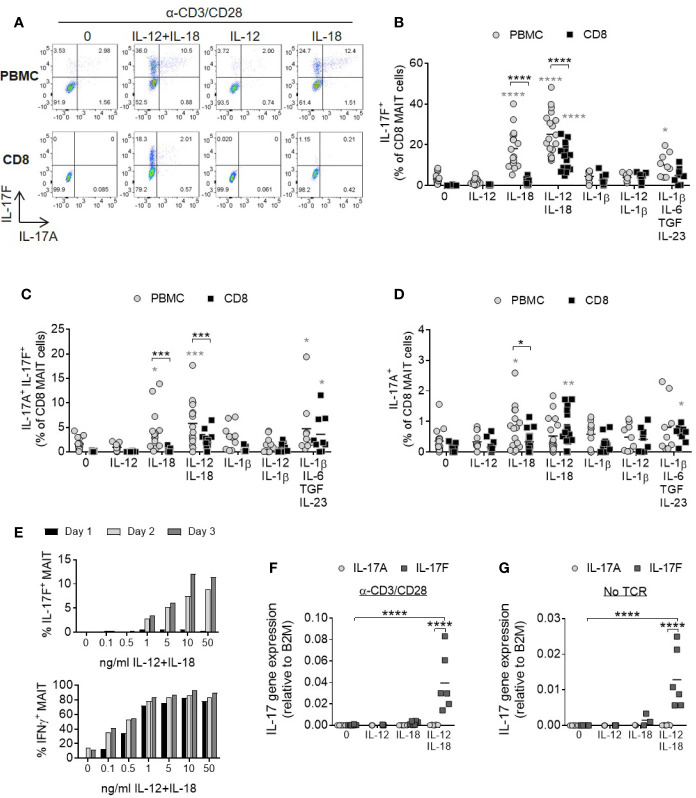
IL-12 and IL-18 signaling promote MAIT cell IL-17A and IL-17 F production. **(A)** Representative bivariate FC plots of PBMCs or purified CD8^+^ T cells cultured for 3 days with anti-CD3/CD28 alone or with additional IL-12, IL-18, or IL-12+IL-18. Brefeldin A was added for 6 h on Day 3. **(B–D)** Combined results of IL-17F^+^ IL-17A^-^, IL-17F^+^ IL-17A^+^, or IL-17F^-^ IL-17A^+^ production from CD8^+^ MAIT cells following TCR stimulation with cytokines shown on x-axis on Day 3. Comparisons between PBMCs (gray circles) and isolated CD8^+^ T cells (black squares) analyzed using repeated measures two-way ANOVA with multiple comparisons (black stars). Significance comparing each individual treatment condition to ‘0’ shown with gray stars. n = 9–18 donors per condition. **(E)** Kinetics of IL-17F and IFNγ production by MAIT cells from isolated CD8^+^ T cells following anti-CD3/CD28 stimulation for 1, 2 or 3 days and varying concentrations of IL-12 and IL-18. Intracellular cytokine production assessed by flow cytometry following 6 h Brefeldin A treatment daily. Graphs show data from one donor, representative of 3 individual experiments. **(F, G)** IL-17A and IL-17F gene expression in isolated CD8^+^ T cells cultured for 3 days with or without anti-CD3/CD28, and with or without IL-12, IL-18, or IL-12+IL-18. Significance measured using two-way ANOVA with multiple comparisons between cytokines/treatment conditions (*n* = 6). *p<0.05, **p<0.01, ***p<0.001, ****p<0.0001.

In a complete cell mixture of PBMCs, addition of IL-18 with or without IL-12 together with TCR stimulation promoted robust IL-17F production from MAIT cells (**Figure 2A**). In contrast, MAIT cell IL-17A and IL-17F production from a purified CD8+ population required both IL-12 and IL-18 cytokines. The smaller populations of CD4+ and CD4-CD8- MAIT cells responded similarly (**Supplementary Figures 4A–D**). Compared to non-MAIT cells, CD161+ Vα7.2+ MAIT cells were the only subset in the T cell compartment showing enhanced IL-17A and IL17-F production in the presence of IL-12 and IL-18 (**Supplementary Figures 5A–D**).

IL-1β has a shared signaling pathway with IL-18 and a role in promoting human Th17 responses. Either alone or together with IL-12, IL-1β did not induce a significant increase in MAIT cell IL-17A or IL-17F production (**Figures 2B–D**). This may be due to receptor expression as MAIT cells express high levels of IL-18Rα but not IL-1 receptors (**Supplementary Figures 6A–C**). A cocktail of cytokines known to be important for Th17 generation (IL-1β, IL-6, IL-23, and TGFβ) (29) also induced MAIT cell IL-17A and IL-17F production, but to a lesser degree than the synergistic combination of IL-12 and IL-18 (**Figures 2B–D**).

IL-17F production by MAIT cells was dose- and time-dependent and showed delayed kinetics relative to IFNγ (**Figure 2E**). IL-17A levels were very low (**Supplementary Figures 7A, B**).

Gene expression analysis of isolated CD8+ T cells showed upregulation of IL-17F mRNA upon stimulation with IL-12 and IL-18, but not either cytokine alone (**Figures 2F, G**). Cells stimulated with cytokines expressed significantly higher levels of IL-17F mRNA compared to IL-17A and this was enhanced further in the presence of TCR stimulation, suggesting IL-12/IL-18 regulate the induction of gene expression rather than *via* post-transcriptional modification of IL-17F. TCR engagement is additionally necessary for optimal induction of IL-17F transcription.

### IL-12 and IL-18-Driven MAIT Cell IL-17A and IL-17F Production Is Independent of IL-23

**Figure 3:**
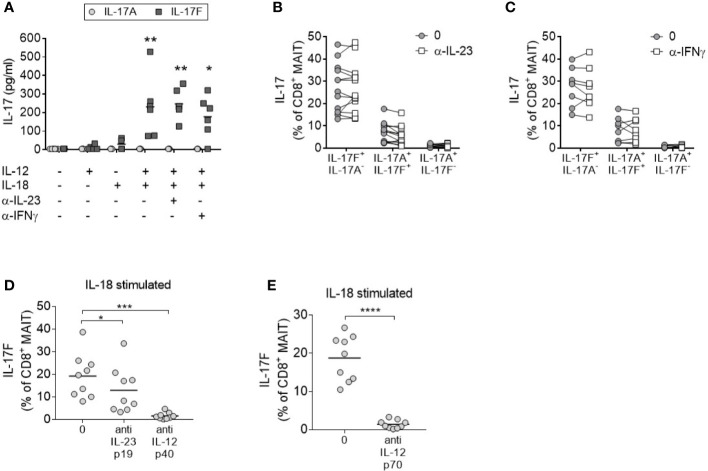
MAIT cell IL-17 production in response to IL-12 and IL-18 is IL-23 independent. **(A)** Levels of IL-17A (light gray circle) or IL-17F (dark gray square) in the culture supernatants from CD8^+^ T cells cultured for 3 days with anti-CD3/CD28 and additional cytokines and blocking antibodies as displayed on the x-axis. Significance measured using two-way ANOVA with multiple comparisons and shown for treatment groups compared to the first ‘-’ group (*n* = 6). **(B–D)** IL-17F^+^ IL-17A^-^, IL-17F^+^ IL-17A^+^, or IL-17F^-^ IL-17A^+^ production by MAIT cells measured by flow cytometry of PBMCs cultured for 3 days with anti-CD3/CD28 and IL-12+IL-18 with or without the addition of neutralizing antibodies to **(B)** IL-23p19 (*n* = 13) or **(C)** IFNγ (*n* = 7) or **(D)**. Statistics measured using two-way ANOVA with multiple comparisons. **(D, E)** Combined results showing IL-17F^+^ IL-17A^-^ production from CD8^+^ MAIT cells following 3 days of anti-CD3/CD28 stimulation of PBMCs with IL-18, and inhibition of either IL-23p19 and IL-12p40 or IL-12p70 (*n* = 9). Statistics measured using repeated measures one-way ANOVA **(D)** or paired t-test **(E)**. *p<0.05, **p<0.01, ***p<0.001, ****p<0.0001.

To evaluate the contribution of IL-23 in IL-17A and IL-17F production by MAIT cells, enriched CD8+ T cells were stimulated in the presence of an anti-IL-23p19 antibody. Blockade of IL-23 signaling did not impair the secretion of IL-17F in response to anti-CD3/CD28 stimulation in the presence of IL-12 and IL-18 (**Figure 3A**). As MAIT cells were the only IL-17F producing CD8+ subset after cytokine stimulation (**Supplementary Figures 5A–D**), all IL-17F present in the supernatants was assumed to be secreted from MAIT cells. Neutralization of IFNγ also had no discernible effect on IL-17A or IL-17F expression.

Consistent with enriched CD8+ T cells, the activation of MAIT cells within complete PBMC cultures promoted IL-17A and IL-17F production independently of IL-23 or IFN*γ* signaling (**Figures 3B, C**). Neutralization of IL-12p40 (**Figure 3D**) or IL-12p70 (**Figure 3E**) in the same assay confirmed the requirement of IL-12 signaling. In contrast to CD8+ MAIT cells, inhibition of IL-23 signaling in CD4+ Th17 cells demonstrated a significant reduction of IL-17A and IL-17F after 3 days (**Supplementary Figure 8**).

### Monocyte-Derived IL-12 Promotes MAIT Cell IL-17A and IL-17F Production in the Presence of IL-18

**Figure 4:**
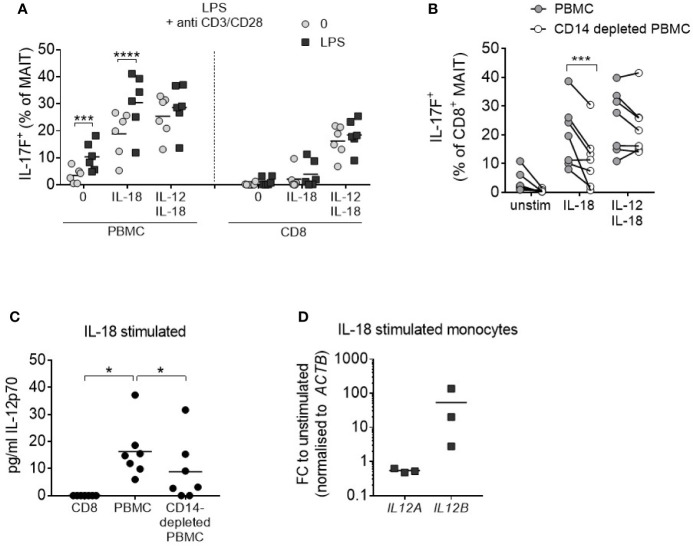
IL-18-induced MAIT cell IL-17A and IL-17F production requires monocytes. **(A)** Frequency of CD8^+^ IL-17F-producing MAIT cells following 3 days of anti-CD3/CD28 stimulation of PBMCs or isolated CD8^+^ T cells with or without IL-18, IL-12 + IL-18, or LPS (10 ng/mL) for the duration of culture (*n* = 6). Two-way ANOVA comparing ‘0’ and ‘LPS’. **(B)** IL-17F production by CD8^+^ MAIT cells from PBMCs or CD14-depleted PBMCs from matched donors following 3-day anti-CD3/CD28 stimulation with or without IL-18 or IL-12 + IL-18. Repeated measures two-way ANOVA with multiple comparisons (*n* = 7). **(C)** IL-12p70 levels in culture supernatant following 3-day anti-CD3/CD28 stimulation + IL-18 comparing isolated CD8^+^ T cells, PBMCs or CD14-depleted PBMCs. Repeated measures one-way ANOVA (*n* = 7). **(D)** *IL12A* and *IL12B* expression in FACS-purified monocytes stimulated for 18 h with IL-18; fold change relative to FACS-purified, unstimulated monocytes, normalized to *ACTB* (*n* = 3). *p<0.05, ***p<0.001, ****p<0.0001.

It is well documented that monocytes produce IL-12 (30), and so these cells were examined as a potential endogenous IL-12 source within PBMC cultures. PBMCs were stimulated with anti-CD3/CD28/IL-18 and the monocyte component was concurrently activated through TLR4 agonism by LPS. This resulted in increased IL-17F production from MAIT cells to levels comparable with the addition of exogenous IL-12/IL-18 (**Figure 4A**). Isolated CD8+ T cells which do not directly respond to LPS showed no change in IL-17F production upon LPS treatment. Culturing PBMCs depleted of CD14+ monocytes with IL-18 alone led to a reduction in IL-17A and IL-17F-production by MAIT cells (**Figure 4B**, **Supplementary Figures 9A, B**), together with a reduction in IL-12 levels (**Figure 4C**). Purified monocytes cultured with IL-18 showed an upregulation in IL-12B (IL-12p40) (**Figure 4D**), confirming the direct impact of this cytokine on IL-12 synthesis.

### MAIT Cell IL-17A and IL-17F Production Upon Bacterial Stimulation Requires MR1, IL-12, and IL-18

**Figure 5:**
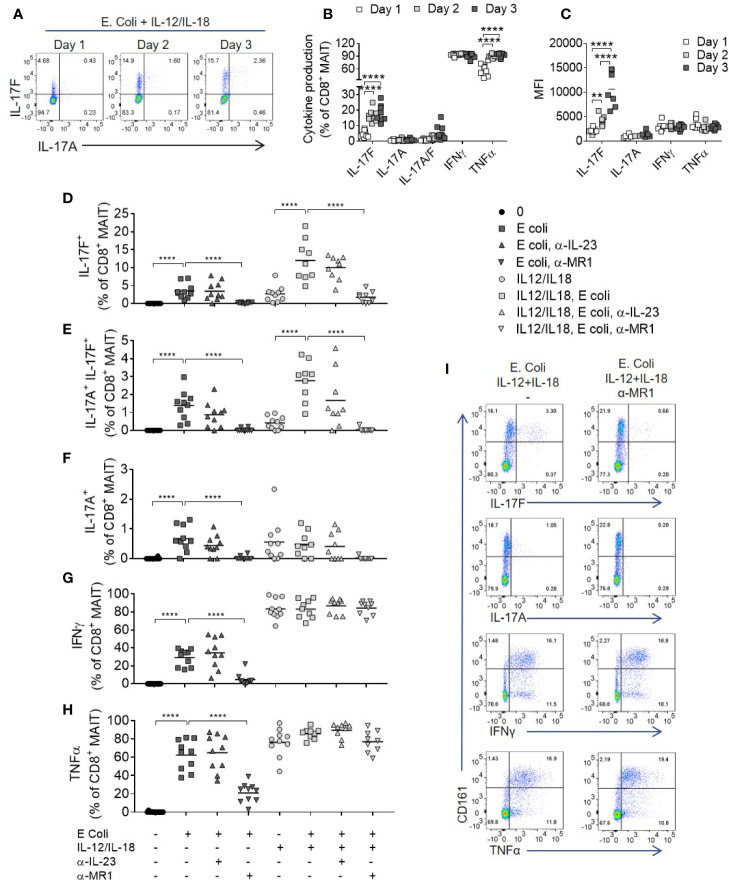
Optimal IL-17A and Il-17F production from MAIT cells requires MR1 and IL-12/IL-18 signaling. **(A)** Representative bivariate FC plots of IL-17F and IL-17A production by MAIT cells following culture of PBMCs with fixed *E. coli* for 1, 2, or 3 days with IL-12 and IL-18. **(B)** Frequency of cytokine producing MAIT cells following 1, 2, or 3 days of PBMC culture with fixed *E. coli*, IL-12, and IL-18. Significance calculated using two-way ANOVA with multiple comparisons across days (*n* = 8). **(C)** Median fluorescence intensity of cytokine producing MAIT cells cultured as in **(B)**, analyzed as the median fluorescence intensity gated on the cytokine producing subset. Significance calculated using two-way ANOVA with multiple comparisons across days (*n* = 8). **(D–H)** Combined results showing the frequency of cytokine producing CD8^+^ MAIT cells following 3 days of culture with fixed *E. coli* with or without IL-12 and IL-18, and with or without the addition of antibodies inhibiting MR1 or IL-23p19. Statistics calculated as two-way ANOVA, with repeated measures using either ‘*E. coli’* or ‘IL-12/IL-18 *E. coli*’ as reference group (*n* = 10). **(I)** Representative bivariate FC plots of PBMCs stimulated with fixed *E. coli*, IL-12 and IL-18 for 72 h, showing cytokine production gated on total CD3^+^ T cells, with or without MR1 blockade. **p<0.01, ****p<0.0001.

In contrast to anti-CD3/CD28 stimulation which broadly activates multiple T cell subsets (**Supplementary Figure 10**), we next sought to test *E. coli* (DH5α strain) as a more natural source of MAIT cell ligands, enabling specific activation of MR1-restricted MAIT cells within PBMCs. Addition of *E. coli* with IL-12 and IL-18 induced a significant proportion of IL-17F-producing MAIT cells (**Figure 5A**), similar to the amount induced by anti-CD3/CD28 stimulation. Optimal IL-17F production occurred after 2–3 days (**Figures 5B, C**), as in polyclonally activated CD8+ T cells (**Figure 2E**). IL-17A and IL-17F did not further increase beyond Day 3 (**Supplementary Figures 11A–D**).

After addition of IL-12 and IL-18, IFNγ and TNF were produced independently of MR1, (**Figures 5D–I**) in contrast to IL-17A and IL-17F, which required both MR1 and IL-12/IL-18 stimulation for optimal production (**Figures 5D–F**). Importantly, MAIT cell IL-17A and IL-17F production induced by *E. coli*, with or without IL-12 and IL-18 stimulation, was IL-23 independent. A low level of TCR independent IL-17A and IL-17F production was observed upon stimulation with IL-12 and IL-18 alone, supporting gene expression results where IL-12 and IL-18 upregulated IL-17F mRNA without a TCR trigger (**Figures 2F, G**). While the addition of IL-12 and IL-18 enhanced MAIT cell cytokine production, all cytokines examined were produced upon *E. coli* stimulation alone without additional IL-12 and IL-18, again being IL-23 independent but MR1 dependent (**Figures 5D–H**).

**Figure 6:**
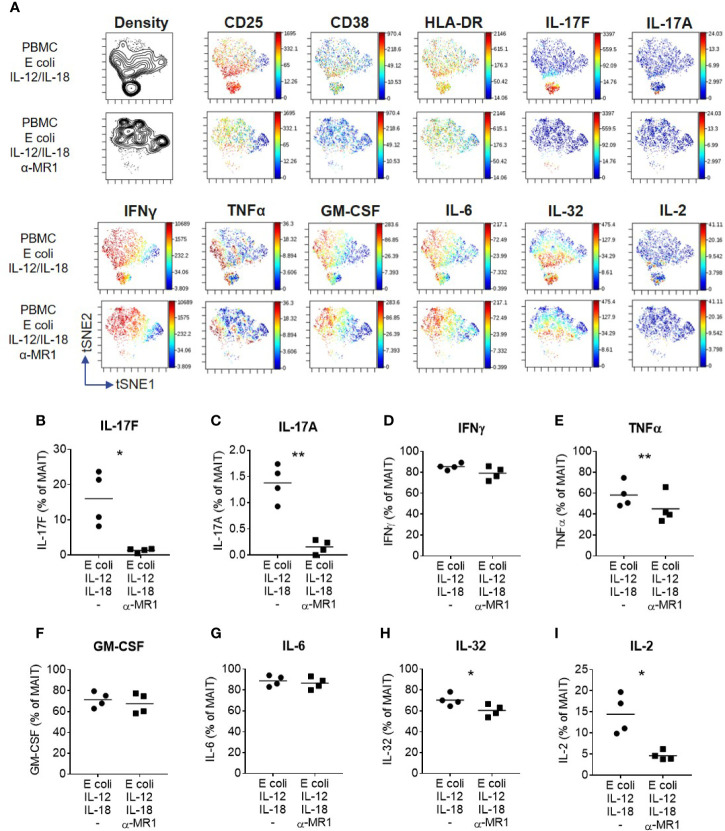
IL-17F producing MAIT cells form a distinct cluster of highly activated cells. **(A)** t-SNE plots from data generated by CyTOF, gated on CD161^+^ Vα7.2^+^ MAIT cells from PBMCs stimulated for 3 days with fixed *E. coli*, IL-12 and IL-18. Plots are made up of concatenated files from four donors, since each individual showed the same trends. Plots show density or relative expression of the indicated markers according to the color scale. Clustering was performed using channels containing cytokine markers. **(B–I)** Percentage of MAIT cells producing cytokines upon stimulation of PBMCs with *E. coli*, IL-12 and IL-18, with or without anti-MR1 blockade. Significance measured using paired t-tests (*n* = 4). *p<0.05, **p<0.01

A wider range of cytokines were investigated using high-dimensional single cell mass cytometry analysis of MAIT cells from PBMCs activated with *E. coli*, IL-12 and IL-18, with or without anti-MR1. viSNE clustering revealed a distinct cluster that was characterized by IL-17F-producing MAIT cells (**Figure 6A**). This cluster expressed high levels of CD25, CD38 and HLA-DR, suggesting highly activated cells. IL-17A and IL-17F were unique in their expression being restricted to this cluster alone, which was almost completely abolished upon MR1 blockade. The pro-inflammatory cytokine IL-32 associated most closely with the IL-17 cluster, albeit also showing polyfunctionality with other cytokines such as IFNγ, GM CSF and IL-6. Several cytokines were undetectable in this system, including IL-10, IL-13, IL-21, IL-22, and IL-8 (**Supplementary Figure 12**). While TNF, IL-32 and IL-2 were partially reduced, IL-17A and IL-17F showed the most complete inhibition upon MR1 blockade (**Figures 6B–I**).

### Blockade of IL-17A and IL-17F Is Required for Optimal Inhibition of Inflammatory Cytokine Production by Dermal Fibroblasts

**Figure 7:**
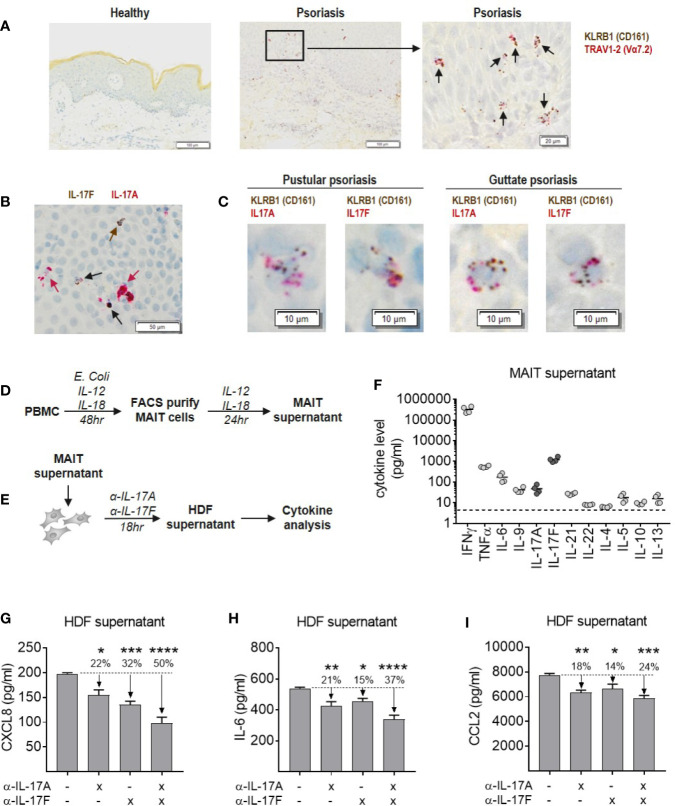
Blockade of IL-17A and IL-17F is required for optimal inhibition of inflammatory cytokine production by dermal fibroblasts in response to MAIT supernatant. **(A)** RNAScope staining of *KLRB1* (brown) and *TRAV1-2* (red) in lesional section from a patient with pustular psoriasis. Co-localization of both probes (black arrows) shows MAIT cells. **(B)** RNAScope staining of *IL-17A* and *IL-17F* in psoriatic lesional skin. *IL-17F* single producing cells (brown arrows), *IL-17A* single producing cells (red arrows) and *IL-17A+IL-17F+* dual producing cells (black arrows). **(C)** RNAScope co-localization of *KLRB1* with *IL-17A* or *IL-17F* in skin sections from two psoriasis patients. **(D)** Experimental setup to generate MAIT cell supernatant. PBMCs were stimulated with *E. coli*, IL-12 and Il-18 for 48 h, after which the activated MAIT cells were purified by FACS and cultured for a further 24 h with IL-2, IL-12, and IL-18. **(E)** Experimental setup to test activity of MAIT cell supernatant in stimulating cytokine release by NHDFs, in the presence or absence of IL-17A and IL-17F neutralization. **(F)** Cytokine levels in MAIT cell supernatant measured using ‘Th cytokine’ 13-plex LegendPlex. IL-2 is not shown as recombinant IL-2 was included in culture media. **(G–I)** Cytokines produced by NHDFs supernatants stimulated with MAIT cell supernatant, with or without neutralizing antibodies towards IL-17A, IL-17F, or IL-17A/IL-17F (bimekizumab). One-way ANOVA with Dunnett’s multiple comparisons, shown relative to ‘0’ group. Background cytokine production from unstimulated fibroblasts were subtracted. *p<0.05, **p<0.01, ***p<0.001, ****p<0.0001.

MAIT cells were abundant in psoriatic lesional skin, identified as cells staining positive for both *KLRB1* (CD161) and *TRAV1-2* (Vα7.2), which were not present in skin from healthy individuals (**Figure 7A**). Isolated CD8+ T cells were used as positive controls for MAIT cell markers to validate staining (**Supplementary Figure 13**). Psoriatic lesional skin contained both IL-17A and IL-17F producing cells, either single positive for IL-17A (red arrow) or IL-17F (brown arrow), as well as IL-17A+IL-17F+ dual-producing cells (black arrow) (**Figure 7B**). In two patients with difficult-to-treat types of psoriasis, we observed both IL-17A and IL-17F-producing KLRB1+ cells (**Figure 7C**). TRAV1-2+ cells were of a much lower abundance (data not shown).

To explore the functional contribution of IL-17A and IL-17F in an *in vitro* model of skin inflammation, activated MAIT cell supernatant was generated (**Figure 7D**), and used to stimulate normal human dermal fibroblasts (NHDFs) in the presence or absence of antibodies neutralizing IL-17A, IL-17F, or bimekizumab (**Figure 7E**). Profiling of MAIT cell supernatant revealed high levels of inflammatory type-1 and type-17 cytokines consistent with CyTOF data, while type-2 cytokines were around the lower limit of detection (**Figure 7F**). Addition of MAIT cell supernatant to NHDFs induced production of pro-inflammatory IL-6, granulocyte-attracting CXCL8 and myeloid cell-attracting CCL2, which were significantly reduced upon IL-17 blockade (**Figures 7G–I**). While blockade of IL-17A or IL-17F alone reduced the production of these cytokines by NHDFs, blockade of both cytokines simultaneously had the greatest inhibitory effect.

### IL-17A and IL-17F Production From Other Innate Lymphocytes Is Biased to IL-17F and Independent of IL-23

Finally, we assessed whether IL-23 independent IL-17A and IL-17F production was shared among other innate lymphocytes. Initial analysis was focused on group 3 ILCs based on literature data suggesting these cells express RORγt and IL-17A (31). To begin with, we examined an enriched population of ILCs and found that IL-17A and IL-17F were produced by ILC3s upon stimulation with IL-1β, IL-2 and IL-7, with or without IL-23, and with a bias towards IL-17F (**Supplementary Figure 14A**). To extend this initial finding, we obtained a pure population of ILC3s by depleting lineage positive cells from PBMCs, followed by cell sorting of CD45+ Lin- CD161+ CD127+ c-kit+ CRTH2- cells (**Supplementary Figure 14B**). In three separate donors, following culture with IL-1β and IL-2, these cells produced both IL-17A and IL-17F. There was a non-significant trend towards increased IL-17A and IL-17F production upon IL-23 treatment, but high levels of IL-17A and IL-17F were still observed after IL-23 blockade (**Supplementary Figure 14C**). As with MAIT cells, the bias towards IL-17F was apparent both at protein and gene level in ILC3s (**Supplementary Figure 14D**), with gene expression data obtained from a further two separate donors. Activated γδ T cells showed the same trend by producing predominantly IL-17F in an IL-23 independent manner (**Supplementary Figures 14E, F**).

## Discussion

Conflicting conclusions have been drawn regarding MAIT cell IL-17A production over recent years, with little known of the factors regulating this function. Here, we show that prolonged stimulation with IL-12 and IL-18, together with TCR triggering, robustly induces IL-17A and more notably IL-17F production by MAIT cells, and that this is independent of IL-23. Distinct differences in the regulation of IL-17A and IL-17F production over other cytokines was most evident following unsupervised viSNE analysis and provides a possible explanation as to why MAIT cell IL-17 has remained elusive. The kinetics of both IL-17A and IL-17F production and dependency on cytokine and TCR stimulation differ from those of IFNγ and TNF, which have been the prototypic readouts for activated MAIT cells. Furthermore, in contrast to much of the previously published work, we did not use PMA and ionomycin to stimulate MAIT cells as this created an artificial bias towards IL-17A that is not reflected in ELISA data. The combination of temporal differences in IL-17F synthesis as well as the methodological modifications we employ in this study may in part explain the dearth of publications on IL-17F, as well as clarify the IL-17A-focused literature on MAIT cells (32).

IL-23 independent production of IL-17A and IL-17F by γδ T cells and ILC3s in addition to MAIT cells suggests that this feature may be shared among innate-like lymphocytes. This is strengthened by a recent study showing entheseal γδ T cells can produce IL-17A independently of IL-23R expression (33). Together, these results may begin to explain the lack of clinical response to IL-23 inhibition in axial spondyloarthritis, a disease where it is becoming increasingly understood that the main source of IL-17A is from innate immune cells (34), and the IL-17A response plays a pivotal role in disease (3, 26). While IL-23 independent IL-17A and IL-17F production from MAIT and γδ T cells required IL-12, ILC3s did not require IL-12 or IL-23. As ILC3s are enriched in spondyloarthritis synovial tissue (34, 35), production of IL-17A and IL-17F by these cells could offer an explanation as to why ustekinumab, which targets the p40 subunit common to both IL-12 and IL-23, was also not efficacious in axial spondyloarthritis (36).

IL-12B mRNA expression has been associated with susceptibility to skin inflammation (37) and was found to be increased in IL-18 stimulated monocytes in this study. Increased IL-18 levels in serum and at inflammatory sites have been noted in several diseases (38). These results provide a possible link between these observations and IL-17A and IL-17F production by MAIT cells. While IL-12 and IL-18 are not prototypic type-17 inducing cytokines, their action on MAIT cells may differ due to the constitutive expression of RORγt. A role for IL-18 in inducing IL-17A production has been proposed in CD4+ and γδ T cells (39), whereas IL-12 typically inhibits IL-17 production from CD4+ T cells while inducing IFNγ (40). An important area of future research will be establishing molecular mechanisms of how these cytokines induce MAIT cell IL-17A and IL-17F.

In contrast to IFNγ and TNF production from MAIT cells which occurred early on, IL-17A and IL-17F production required prolonged stimulation. Similarly, Böttcher et al. found that MAIT cells produce IL-17A after stimulation with IL-12 and IL-18 for 72 h, but not 24 h (41). Based on our findings, it is likely that many of the IL-17A cells identified by Böttcher et al. were also producing IL-17F. Time-dependent differences in MAIT cell activation are further supported by a recent report comparing MAIT cell activation with purified antigens from *E. coli* extracts at 24 or 72 h, showing greater activation markers at 72 h (42).

IL-17A and IL-17F expressing MAIT cells were unique in their cytokine producing profile and dependency on MR1. This unique profile suggests tighter regulation of MAIT cell production of IL-17A and IL-17F relative to other cytokines, as other cytokines could be produced independently of MR1 following IL-12 and IL-18 signaling. IL-32, a cytokine that can exacerbate inflammation (43), is associated most closely with IL-17A and IL-17F-producing MAIT cells, supporting this population of cells as highly pro-inflammatory. A recent study showed that TCR-triggered MAIT cells may be involved in tissue repair processes, while pathological processes are induced upon activation with both TCR and cytokine stimuli (44) further supporting the heightened inflammatory potential upon combined TCR and cytokine activation.

A key role for monocytes in MAIT cell IL-17A and IL-17F production was identified. Only MAIT cells from TCR-triggered PBMCs, but not isolated CD8+ T cells, produced IL-17A and IL-17F upon stimulation with IL-18 alone. As antigen presenting cells induce MAIT cell IFNγ production *via* IL-12 and IL-18 independently of TCR signals (19, 45), and IL-12 is primarily produced from myeloid cells (30), we explored the dependency on IL-12 in our system. Here, we demonstrate that IL-18 directly stimulates monocyte IL-12 production, enabling MAIT cells to produce IL-17A and IL-17F in addition to IFNγ and TNF.

Surprisingly, IL-17F was the dominant isoform produced by all innate lymphocytes, including MAIT cells, γδ T cells and ILC3s. These observations suggest IL-17A and IL-17F may be differentially regulated cytokines. Indeed, STAT5 and TNFAIP3 have been shown to differentially influence the production of these two related isoforms (46–48). These data may explain why IL-17F is also the dominant IL-17 isoform expressed in inflammatory diseases such as psoriasis, psoriatic arthritis and ankylosing spondylitis where IL-17F levels are measured to be at least 30-fold higher than IL-17A (49). Despite its reduced potency relative to IL-17A, our previous work focusing on Th17 cells (24) showed a clear contribution of IL-17F to inflammation. We now extend this work and demonstrate *in vitro* dual inhibition of IL-17A and IL-17F is required to fully suppress IL-17 driven inflammation by activated MAIT cells. Whether MAIT cells are drivers of pathogenesis during inflammation still requires investigation; however, their contribution has been suggested in a number of disease indications, including in recent clinical trials in ankylosing spondylitis (2) and psoriatic arthritis (26). We identified KLRB1 expressing cells in psoriatic lesional skin that produced both IL-17A and IL-17F, although we were unable to determine the IL-23 dependency in these samples. The lack of TRAV1-2 cells expressing IL-17A and IL-17F could be due to TCR downregulation that accompanies MAIT cell activation (50). Nevertheless, the abundance of both IL-17A and IL-17F expression in psoriatic lesional skin originating from CD161+ cells, in the context of our *in vitro* data showing the contribution of both of these cytokines to inflammatory responses, suggests a role for both IL-17A and IL-17F in disease pathogenesis. This has potential clinical applications, as patients showing high IL-23 independent production of IL-17A and IL-17F may be better served by therapeutics neutralizing IL-17A and IL-17F directly, rather than IL-23 inhibition. This could also identify or explain potential clinical non-responders, which would enable treatment with the most appropriate therapeutic in the first instance. In contrast to other published reports, RNAscope analysis of healthy skin samples detected very few, if any, MAIT cells. However, given the low frequencies of MAIT cells in healthy skin (51), it is likely that the RNAScope technique, which only shows a cross-section of a small skin area, does not capture these rare cells.

A potential limitation of our study is that our *in vitro* focus on peripheral blood-derived human MAIT cells precluded investigation into the role of IL-23 in MAIT cell development or function in human tissues. Our study focused primarily on human cells for three reasons. Firstly, the potency of IL-17F in mice is approximately 100 times weaker than that of human IL-17F when tested on human cells. Secondly, given the rarity of MAIT cells in mice we decided to focus on developing human models of skin activation to better understand the inflammatory potential of IL-17F relative to IL-17A. Lastly, to investigate the relevance of MAIT derived IL-17A and IL-17F to inflammatory disease we complimented our *in vitro* data with RNAscope analysis of MAIT cells in human psoriatic lesional skin. Functional phenotype differences have been identified between circulating and tissue resident MAIT cells, with MAIT cells also expressing a tissue resident gene expression profile (52). MAIT cells in lung tissue have been shown to be more prone to IL-17A and IL-17F production than their blood counterparts (53). Analysis of BAL fluid in children with pneumonia revealed IL-17F to be more abundantly expressed than IL-17A (52). While we were unable to compare IL-17 responses between blood and tissue resident MAIT cells from the same individuals, this is very much an active area of research, as is understanding whether the requirement for IL-23 independent IL-17 production is similar between tissue compartments. Interestingly, Gracey E et al. (14) report that synovial fluid MAITs from axSpA patients produce IL-17 in response to IL-7 but not in response to IL-23.

Differences between the activation profiles of *in vitro* and *in vivo* MAIT cells have been acknowledged by others (54, 55), where IL-18 deficient mice showed no difference in response to infection *in vivo*, while we and others have observed that IL-18 can drive MAIT cell activation independently of TCR signals *in vitro*. These species differences in MAIT cell activation, observed by us and others, may be due to the specific pathogen-free environment which laboratory mice are housed in, particularly since optimal MAIT cell activation is mediated by bacterial and fungal ligands bound to MR1. It is possible that human MAIT cells are primed in a way that mouse MAIT cells are not, due to previous infections, and therefore have a different activation threshold. Similarly, though relating to IL-23 dependency, it is possible that MAIT cells in humans require IL-23 during their initial activation, and therefore the peripheral MAIT cells in our study have already been primed. This is supported by *in vivo* mouse studies showing a requirement for IL-23 in MAIT cell development and activation (54, 56). We do not suggest that IL-23 does not play an important role in MAIT cell development and activation, but propose alternative pathways to induce the IL-17A and IL-17F in established human MAIT cells. Furthermore, from a translational perspective, our data support the use of downstream IL-17A/IL-17F blockade over IL-23 blockade in IL-17 mediated diseases, as any IL-23 priming will have already occurred by the time patients are treated with therapeutics.

In conclusion, MAIT cells, γδ T cells and innate lymphoid cells produce IL-17A and IL-17F independently of IL-23. Together with the distinct bias towards IL-17F production, this may suggest that dual neutralization of IL-17A and IL-17F could offer advantages over specific IL-17A or IL-23p19 inhibitors for the treatment of IL-17-mediated inflammatory diseases.

## Data Availability Statement

The original contributions presented in the study are included in the article/**Supplementary Material**. Further inquiries can be directed to the corresponding author.

## Ethics Statement

Ethical review and approval was not required for the study on human participants in accordance with the local legislation and institutional requirements. The patients/participants provided their written informed consent to participate in this study.

## Author Contributions

SC and AM conceived the project. SC, JM, RO, and CS carried out experiments. KT contributed towards reagent generation. MG, DB, RO, SS, and AM provided intellectual support. SC drafted the manuscript and figures with subsequent support from all authors. AM supervised the project. All authors contributed to the article and approved the submitted version.

## Funding

The work described here was funded by UCB Pharma.

## Conflict of Interest

All authors are employees of UCB Pharma. CS, KT, DB, SS and AM are shareholders of UCB Pharma. DB has worked as a paid consultant for, and received financial grants from, AbbVie, Pfizer, MSD, Roche, Novartis, Eli Lilly, Boehringer Ingelheim, Glenmark and UCB Pharma.
