# Supplementary material for: Interleukin (IL)-12 and IL-18 Synergize to Promote MAIT Cell IL-17A and IL-17F Production Independently of IL-23 Signaling
Source: Front Immunol. 2020 Nov 20;11:585134. doi: 10.3389/fimmu.2020.585134 (PMC7714946; doi:10.3389/fimmu.2020.585134)
Supplement: Supplementary file 1 [file Table_1.docx]

Supplementary Material

# Supplementary Figures


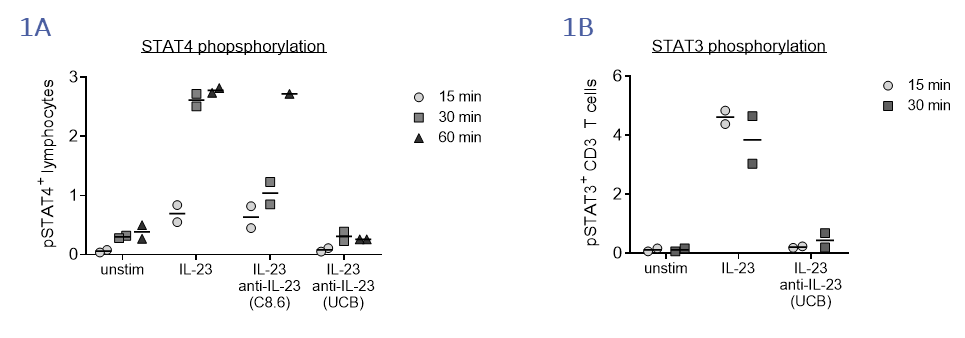


**Supplementary Figure 1: IL-23 signaling pathway is fully inhibited with UCB generated anti-IL-23 antibody.** PBMCs were stimulated with recombinant IL-23 (10ng/mL) in the presence or absence of neutralizing IL-23 antibodies. Phosphorylation of pSTAT4 **(A)** and pSTAT3 **(B)** was examined by flow cytometry after 15, 30 and 60 minutes post stimulation in CD3^+^ gated lymphocytes.


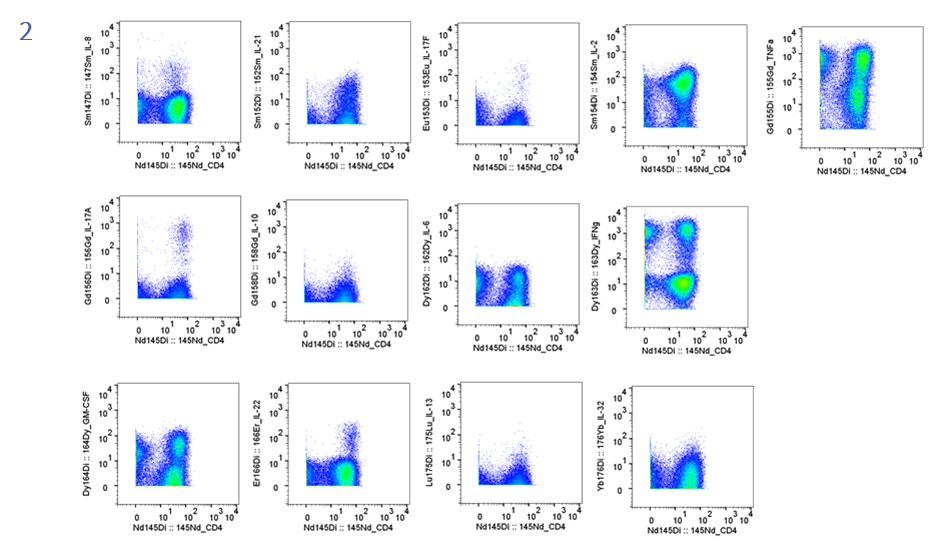


**Supplementary Figure 2: positive controls for CyTOF.** Positive cytokine staining controls for CyTOF^®^ antibodies. PBMCs were stimulated for 4 hours with PMA, ionomycin and Brefeldin A prior to staining.


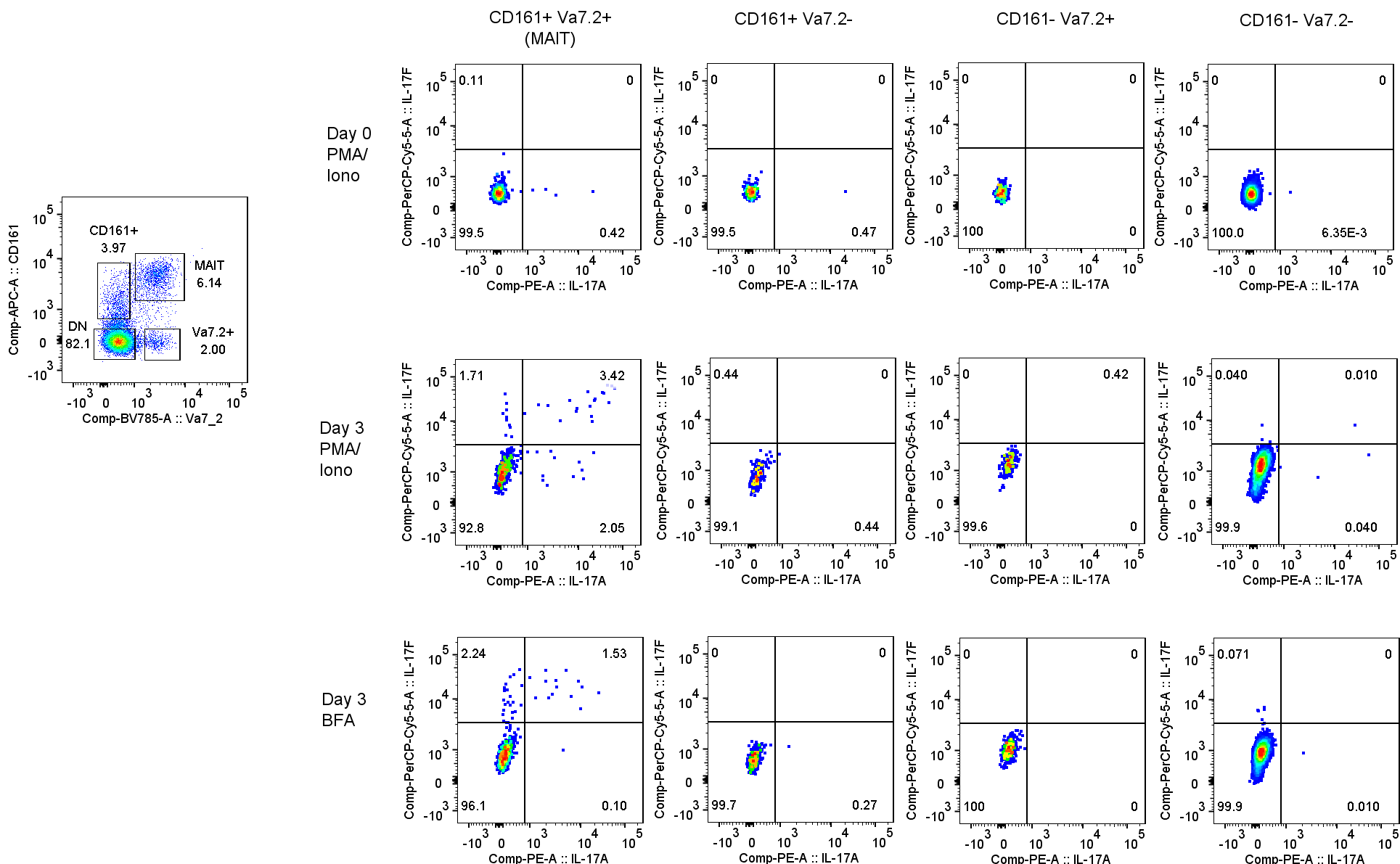


3

**Supplementary Figure 3: Only CD161^+^Vα7.2^+^ MAIT cells produce IL-17A and IL-17F within CD8 T cell compartment.** Resting (Day 0) or anti-CD3/CD28 activated (Day 3) PBMCs were stimulated with PMA and ionomycin on day 0 or day 3. To determine native cytokine production from activated PBMCs samples were also treated with BFA only for the last 4 h of culture. ICS staining of IL-17A and IL-17F expression was assessed in gated CD8 T cells based on the expression of CD161 and Vα7.2 (CD161^+^Vα7.2^+^, CD161^+^Vα7.2^-^, CD161^-^Vα7.2^+^ or CD161^-^Vα7.2^-^)**.**


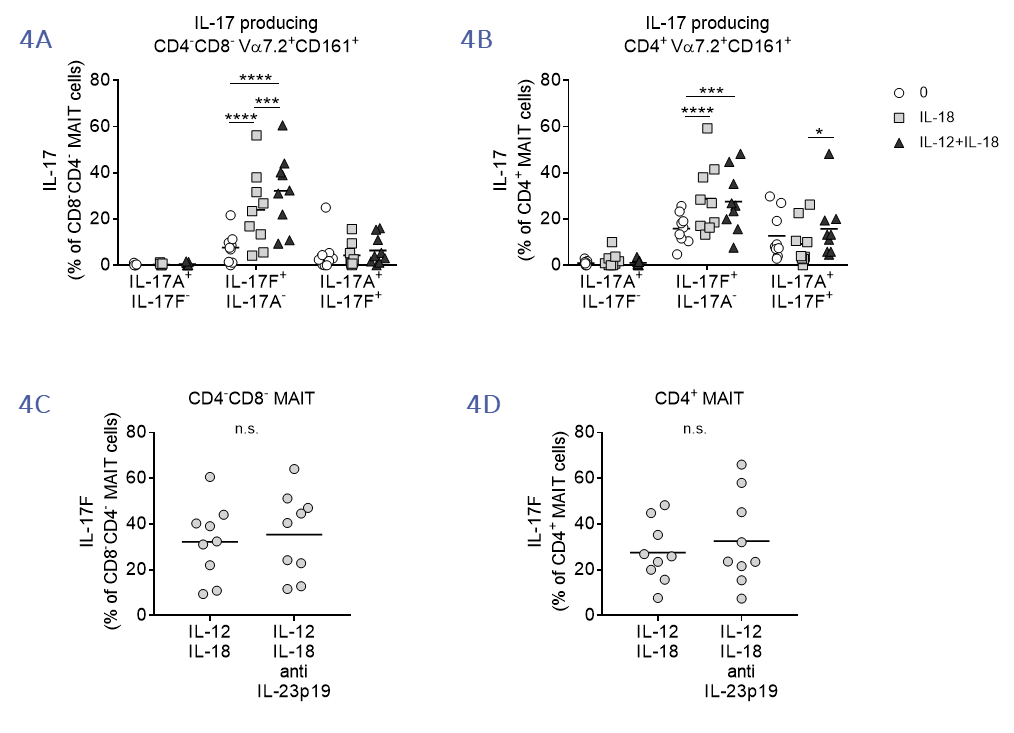


**Supplementary Figure 4: IL-17A and IL-17F production from CD4^+^ and CD4^-^CD8^-^ MAIT cells.** Combined results from *n*=9 donors showing that **(A)** CD4^-^CD8^-^ and **(B)** CD4^+^CD8^-^ MAIT cells (Vα7.2^+^ CD161^++^) also produce high levels of IL-17F following 3 days of anti-CD3/CD28 stimulation +IL-18 +/- IL-12 (two-way ANOVA). **(C-D)** IL-17 production by CD4^-^CD8^-^ and CD4^+^CD8^-^ MAIT cells is IL-23 independent (paired t-test).


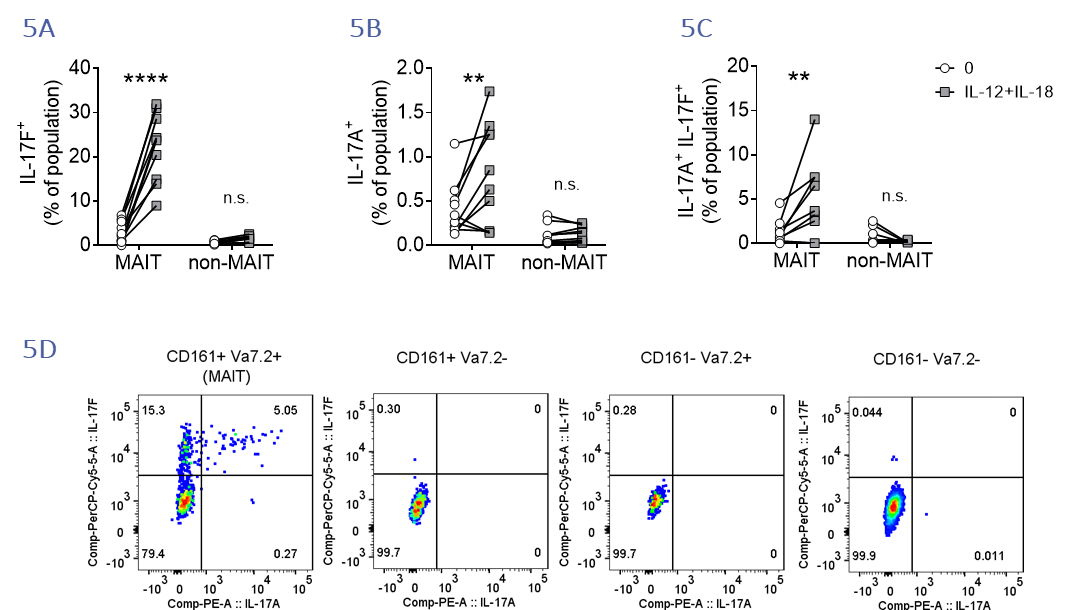


**Supplementary Figure 5: comparison of MAIT and non-MAIT IL-17A and IL-17F production. (A–C)** Frequency of CD3^+^ CD161^+^ Vα7.2^+^ MAIT cells or non-MAIT CD3^+^ T cells producing IL-17F^+^IL-17A^-^, IL-17F^-^IL-17A^+^ or IL-17F^+^IL-17A^+^ following stimulation of PBMCs with anti-CD3/CD28 for 3 days with or without addition of IL-12 and IL-18 (two-way ANOVA). The non-MAIT cells include CD161- Vα7.2-, CD161+Vα7.2- and CD161-Vα7.2+ cells. **(D)** Representative flow cytometry plots showing IL-17A and IL-17F production from CD161+ Vα7.2+ MAIT cells, CD161+ Vα7.2- non-MAIT cells, CD161- Vα7.2+ non-MAIT cells and CD161- Vα7.2- non-MAIT cells.


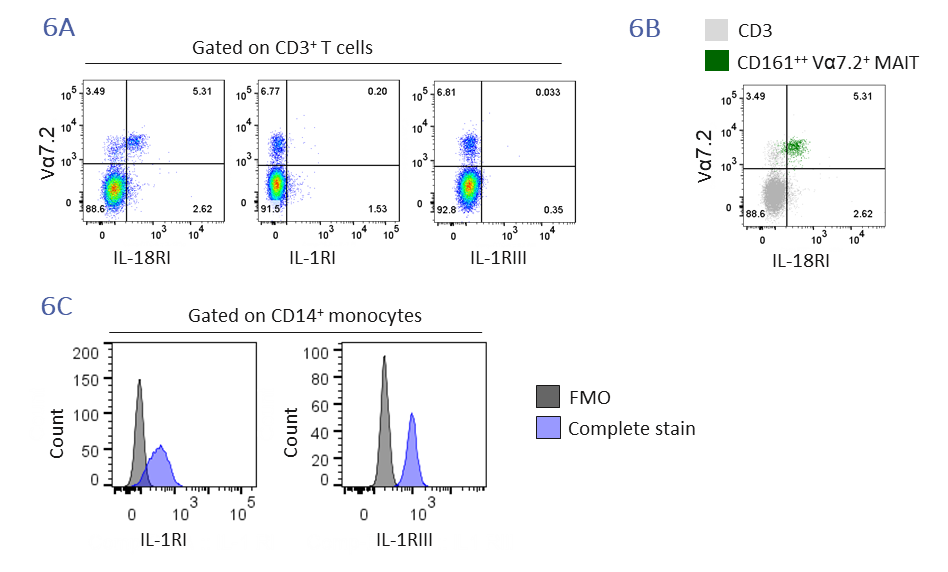
 **Supplementary Figure 6: IL-1 and IL-18 receptor expression. (A)** Flow cytometry staining of PBMCs directly ex vivo showing expression of IL-18RI, IL-1RI, IL-1RII and IL-1RIII on Vα7.2^+^ MAIT cells, gated on CD3^+^ T cells. **(B)** Back-gating of MAIT cells to show their expression of IL-18RI. **(C)** Positive staining of IL-1RI and IL-1R-III on monocytes from the same samples.

***
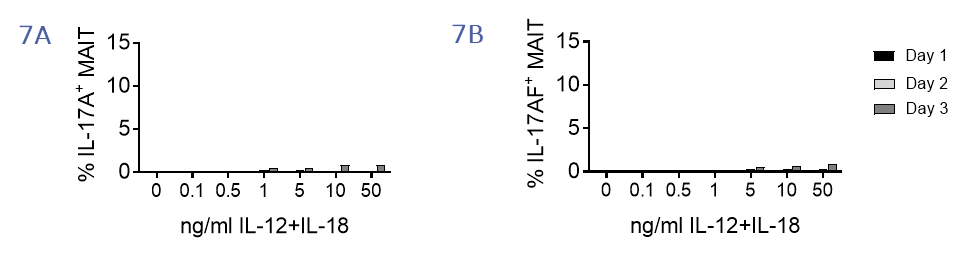
***

**Supplementary Figure 7: IL-17A and IL-17AF production response kinetics to IL-12/IL-18 titration. (A–B)** Daily kinetics of IL-17A^+^IL-17F^-^ and IL-17A^+^IL-17F^+^ production by MAIT cells from isolated CD8^+^ T cells following anti-CD3/CD28 stimulation for 1, 2 or 3 days and varying concentrations of IL-12 and IL-18. Cytokine production assessed by flow cytometry following 6-hour Brefeldin A treatment on each day. Graphs show data from one donor, representative of 3 individual experiments.

*
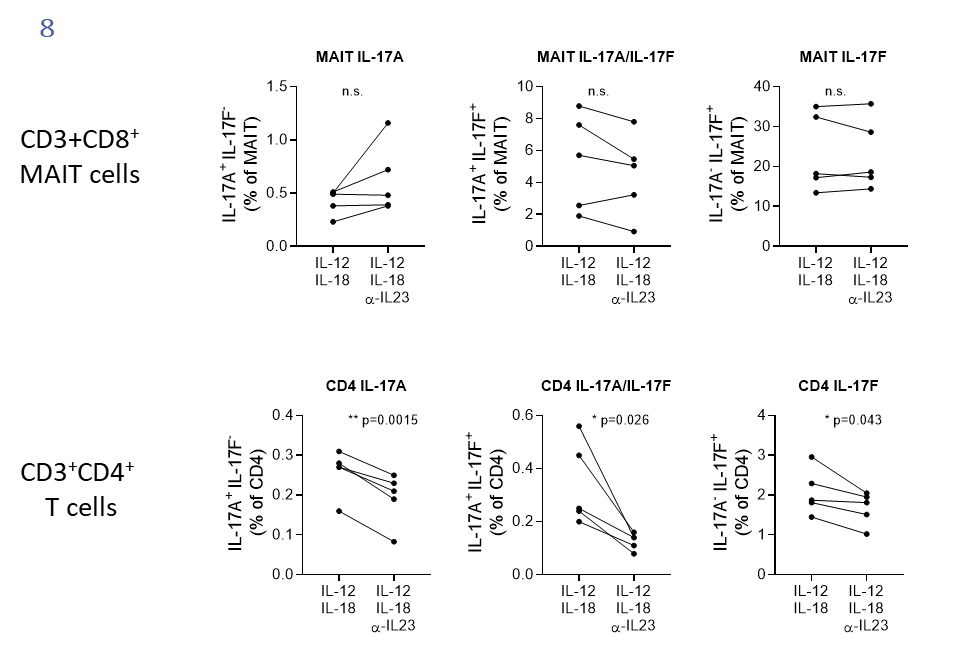
*

**Supplementary Figure 8:** **IL-23 inhibition limits IL-17A and IL-17F in Th17 compartment.** PBMC were activated with anti-CD3/CD28 and recombinant IL-12/IL18 (10ng/mL) for 3 days, in the presence or absence of UCB Pharma’s IL-23 neutralizing antibody. IL-17A and IL-17F expression was assessed in either CD4^+^ T cells or CD8^+^CD161^+^Va7.2^+^ MAIT cells.

***
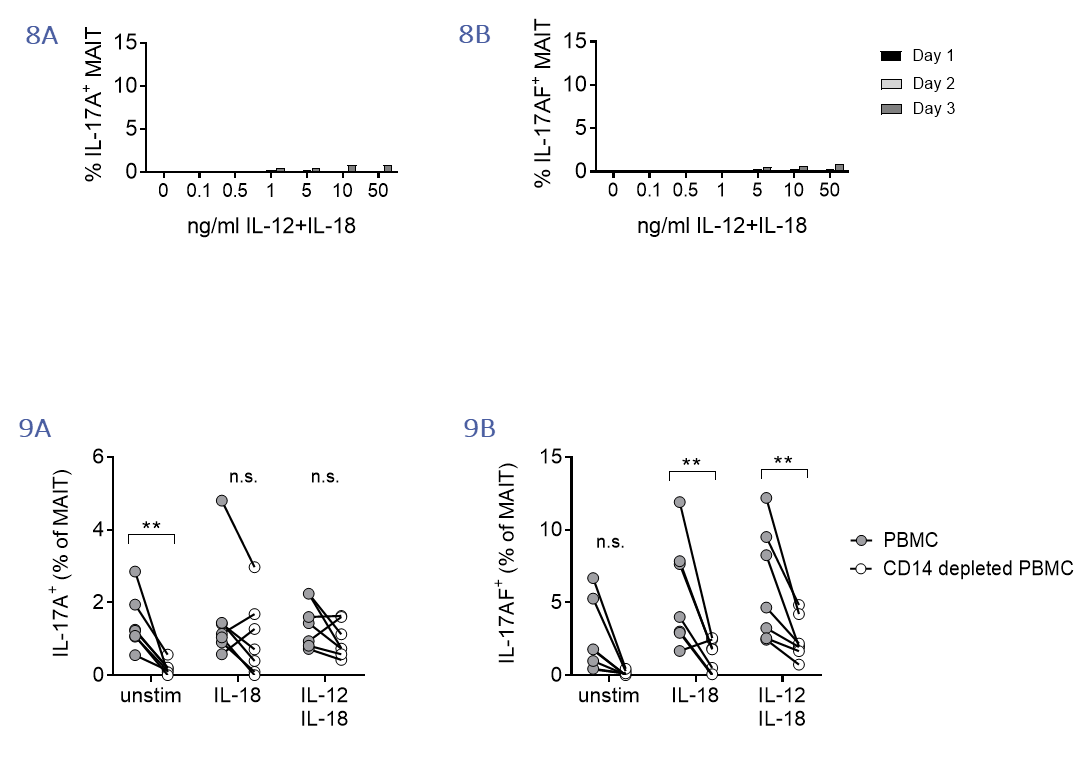
***

**Supplementary Figure 9:** **PBMCs depleted of CD14+ monocytes with IL-18 alone led to a reduction in IL-17A and IL-17F-production by MAIT cells. (A-B)** IL- IL-17A+IL-17F- and IL-17A+IL-17F+ production by CD8+ MAIT cells from PBMCs or CD14-depleted PBMCs from matched donors following 3-day anti-CD3/CD28 stimulation with or without IL-18 or IL-12+IL-18. Statistics measured using repeated measures two-way ANOVA with multiple comparisons between PBMC and CD14-depleted PBMC groups. N=7 donors.

**
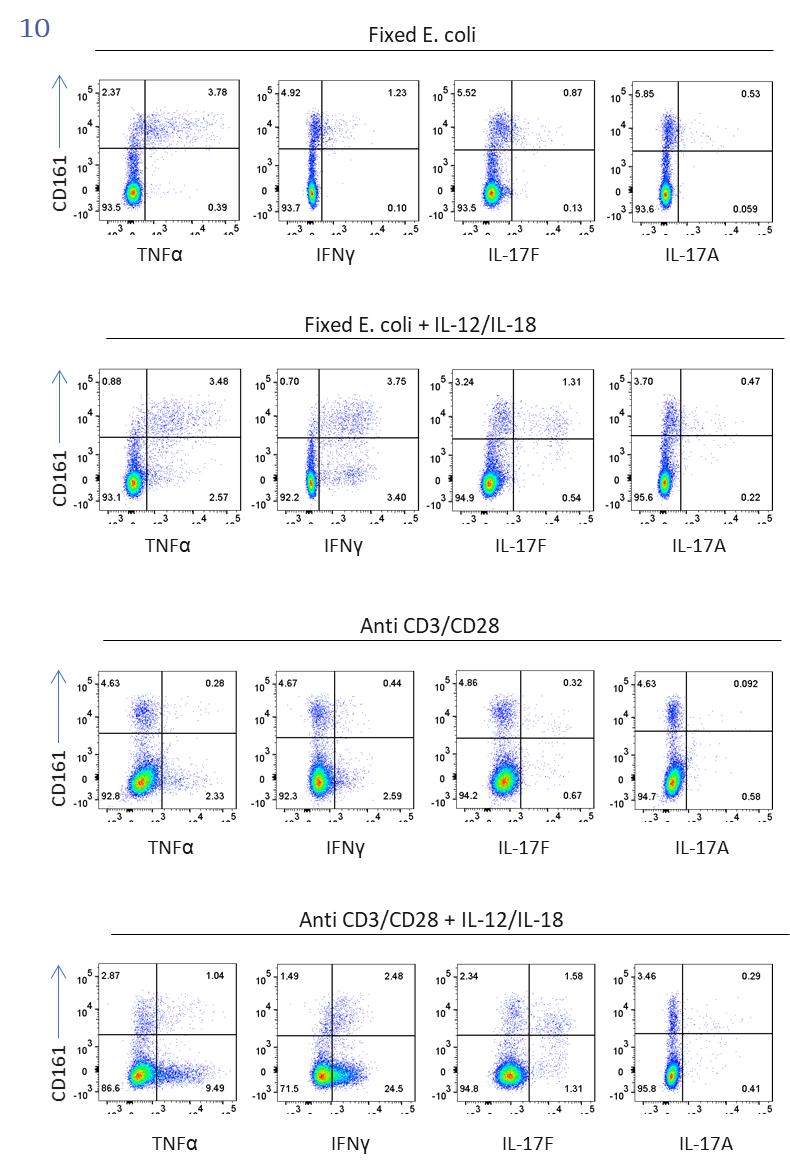
Supplementary Figure 10:** **E coli stimulation preferentially activates CD161^+^ cells.** Comparison of cytokines production from PBMCs stimulated for 3 days with either fixed E. coli or anti-CD3/CD28, with or without IL-12 and IL-18. Cytokine production assessed by flow cytometry following 6-hour Brefeldin A treatment, gated on CD3+ T cells.


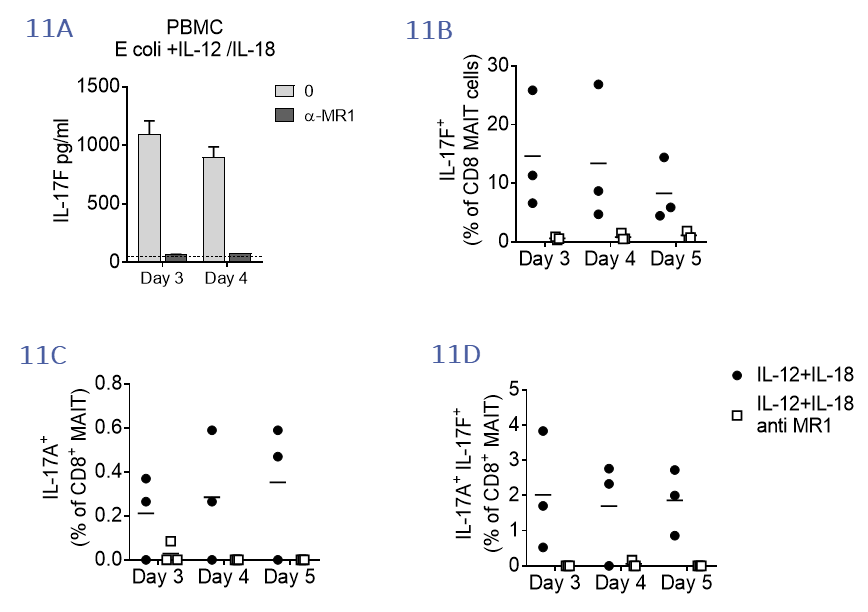


**Supplementary Figure 11: MAIT cell MR1-dependent IL-17A and IL-17F production kinetics. (A)** Level of IL-17F in supernatants from PBMCs stimulated with fixed *E. coli*, IL-12 and IL-18 for 3 or 4 days, with or without anti-MR1 blocking antibody measured by ELISA. **(B-D)** Frequency of IL-17F^+^IL-17A^-^, IL-17F^-^IL-17A^+^ or IL-17F^+^IL-17A^+^ MAIT cells following stimulation of PBMCs with fixed *E. coli*, IL-12 and IL-18 for 3, 4 or 5 days, with or without anti-MR1 blocking antibody.

***
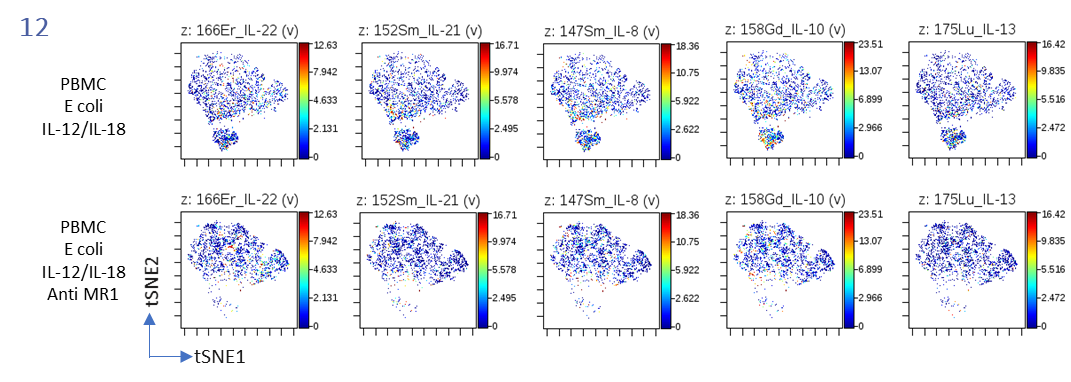
***

**Supplementary Figure 12: t-sne plots showing cytokines not expressed by activated MAIT cells.** t-SNE plots from data generated by CyTOF®, gated on CD161^+^ Vα7.2^+^ MAIT cells from PBMCs stimulated for 3 days with fixed *E. coli,* IL-12 and IL-18. Plots are made up of concatenated files from three donors, since each individual showed the same trends. Plots show relative expression of the indicated markers according to the color scale and display little or no production of IL-22, IL-21, IL-8, IL-10 or IL-13. Clustering was performed using channels containing cytokine markers.


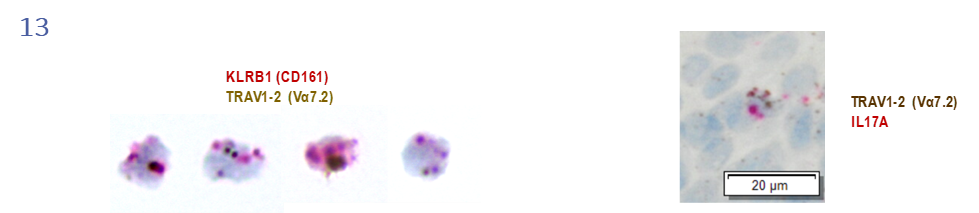


**Supplementary Figure 13: Positive RNAScope controls on isolated CD8+ T cells, showing dual staining of KLRB1 and TRAV1-2 MAIT cells.**

**
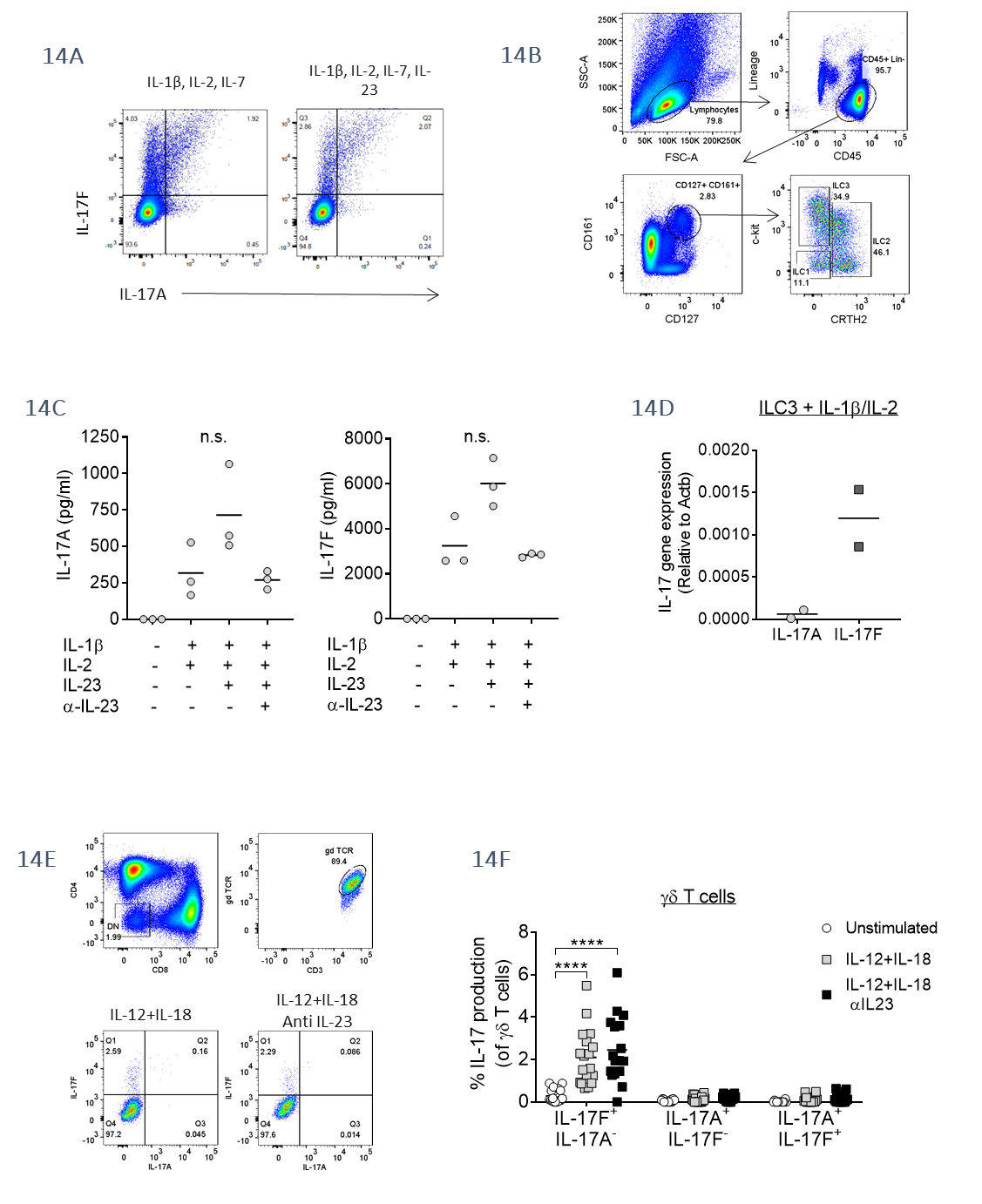
Supplementary Figure 14: ILC and γδ T cells produce IL-17A and IL-17F independently of IL-23 with a bias to IL-17F.** (**A**) Stimulation of enriched ILCs for 7 days with IL-1β, IL-2 and IL-7 with or without IL-23. Cytokine expression examined on day 7 after Brefeldin A treatment and gating on ILC3 cells (Lineage- CD161+ c-kit+ CRTH2-). (**B**) ILCs were purified from PBMCs as lineage negative CD45+ CD127+ CD161+, and subpopulations found according to expression of c-kit and CRTH2. (**C**) IL-17A and IL-17F levels in supernatant from purified ILC3 cultured for 7 days with a combination of IL-1β and IL-2, with or without IL-23 or anti IL-23, n=3 donors. (**D**) Gene expression of IL-17A and IL-17F in FACS purified ILC3 stimulated for 7 days with IL-1β and IL-2, n=2 donors. (**E**) Gamma delta T cells were identified as CD3+ CD4- CD8- γδ TCR+ cells. (**F**) IL-17A and IL-17F expression by gamma delta T cells measured following 3-day stimulation of PBMCs with anti-CD3/CD28 with IL-12 and IL-18, with or without anti IL-23. Two-way ANOVA (n=17).

# Supplementary Table

# Supplementary Table 1. List of antibody metal conjugates used in CyTOF analysis.

| **Metal Antibody Conjugate** | **Target** | **Clone** |
| --- | --- | --- |
| 89Y | CD45 | HI30 (Fluidigm) |
| 103Rh | Viability | - |
| 142Nd | CD19 | HIB19 |
| 143Nd | Va7.2 | 3C10 |
| 145Nd | CD4 | RPA-T4 |
| 146Nd | CD8a | RPA-T8 |
| 147Sm | IL-8 | G265-8 |
| 148Nd | CD16 | 3G8 |
| 149Sm | CD25 | M-A251 |
| 152Sm | IL-21 | 3A3-N2 |
| 153Eu | IL-17F | SHLR17 |
| 154Sm | IL-2 | 1-17H12 |
| 155Gd | TNFa | Mab1 |
| 156Gd | IL-17A | eBio64CAP17 |
| 158Gd | IL-10 | JES3-9D7 |
| 160Gd | CD14 | 61D3 |
| 161Dy | CD161 | DX12 |
| 162Dy | IL-6 | MQ2-13A5 |
| 163Dy | IFNg | 45-15 |
| 164Dy | GM-CSF | BVD2-21C11 |
| 166Er | IL-22 | IL22JOP |
| 167Er | CD27 | 323 |
| 169Tm | CD45RA | HI100 |
| 170Er | CD3 | UCHT1 |
| 171Yb | CD45RO | UCHL1 |
| 172Yb | CD38 | HIT2 |
| 173Yb | CD56 | CMSSB |
| 174Yb | HLA-DR | L243 |
| 175Lu | IL-13 | JES10-5E2 |
| 176Yb | IL-32 | KU32-52 |
| 209Bi | CD11b | ICRF44 (Fluidigm) |
